# Antibacterial Properties of the Antimicrobial Peptide Gallic Acid-Polyphemusin I (GAPI)

**DOI:** 10.3390/antibiotics12091350

**Published:** 2023-08-22

**Authors:** Olivia Lili Zhang, John Yun Niu, Iris Xiaoxue Yin, Ollie Yiru Yu, May Lei Mei, Chun Hung Chu

**Affiliations:** 1 Faculty of Dentistry, The University of Hong Kong, Hong Kong 999077, China; zhlili@connect.hku.hk (O.L.Z.); niuyun@hku.hk (J.Y.N.); irisxyin@hku.hk (I.X.Y.); ollieyu@hku.hk (O.Y.Y.); 2 Faculty of Dentistry, The University of Otago, Dunedin 9054, New Zealand; may.mei@otago.ac.nz

**Keywords:** antimicrobial, caries, peptides, prevention

## Abstract

A novel antimicrobial peptide, GAPI, has been developed recently by grafting gallic acid (GA) to polyphemusin I (PI). The objective of this study was to investigate the antibacterial effects of GAPI on common oral pathogens. This laboratory study used minimum inhibitory concentrations and minimum bactericidal concentrations to assess the antimicrobial properties of GAPI against common oral pathogens. Transmission electron microscopy was used to examine the bacterial morphology both before and after GAPI treatment. The results showed that the minimum inhibitory concentration ranged from 20 μM (*Lactobacillus rhamnosus*) to 320 μM (*Porphyromonas gingivalis*), whereas the minimum bactericidal concentration ranged from 80 μM (*Lactobacillus acidophilus*) to 640 μM (*Actinomyces naeslundii*, *Enterococcus faecalis*, and *Porphyromonas gingivalis*). Transmission electron microscopy showed abnormal curvature of cell membranes, irregular cell shapes, leakage of cytoplasmic content, and disruption of cytoplasmic membranes and cell walls. In conclusion, the GAPI antimicrobial peptide is antibacterial to common oral pathogens, with the potential to be used to manage oral infections.

## 1. Introduction

Oral diseases are a global public health problem affecting over 3.5 billion people worldwide [1]. They can start in early childhood and progress throughout adolescence, adulthood, and old age [2]. Oral diseases have substantial negative effects on individuals, communities, and the wider society. The global economic burden of dental diseases amounts to more than USD 442 billion yearly [3]. The most prevalent oral diseases are dental caries and periodontal disease, which, when left untreated, can progress to tooth loss [4].

Dental caries and periodontal disease are infections resulting from the mixed biofilm (dental plaque) on teeth and periodontal tissues. Dental caries is the localised destruction of dental hard tissue, resulting from the acids that are produced from the sugar fermentation induced by bacteria [5]. *Streptococcus*, *Lactobacillus*, and *Actinomyces* are considered to be the primary cariogenic bacteria involved in the development of dental caries [6]. Pulp and periapical diseases are the secondary diseases of caries; pulpal inflammation and infection usually occur when the pulp is exposed to bacteria. *Enterococcus faecalis* is one of the most frequently found bacteria in teeth with pulp necrosis [7]. Periodontal disease is caused by bacteria, including *Porphyromonas gingivalis* and *Aggregatibacter actinomycetemcomitans* [8]. Thus, biofilm control is the key point for the treatment of oral diseases, such as caries and periodontal disease.

Highly effective antibacterial therapy for caries, endodontics, and periodontics should be applied to achieve optimal outcomes. It is well known that antibiotics are frequently prescribed for pathogens worldwide [9]. However, antibiotics are not clinically used to control cariogenic microorganisms [10]. Many systemic antibiotics, such as penicillin and tetracyclines, do not target oral bacteria specifically [11]. In addition, most antibiotics have side effects, especially for patients who are sensitive to chemical agents, including hypersensitivity and diarrhoea [12]. 

Furthermore, the spread of antibiotic resistance is the greatest problem in using antibiotics [11].The World Health Organization (WHO) has reported that antibiotic resistance is one of the three greatest threats to public health [13]. Oral bacteria tend to be resistant to antibiotics, thus reducing antibiotics’ efficacy [14]. In addition, alarms have been raised concerning the extensive use of adjunctive antibiotics to treat periodontal disease [15]. The European Federation of Periodontology called for a reasonably restrictive and judicious management of adjunctive systemic antibiotics [16]. 

Chlorhexidine is another active bactericidal agent that remains the gold standard of antibiofilm agents. However, it can cause genotoxicity and induce cellular apoptosis [17]. For long-term use, patients frequently report loss of taste, numbness, and extrinsic tooth staining. In endodontics, chlorhexidine is used as a root canal irrigant. However, it is ineffective at dissolving necrotic tissue [18]. In addition, low-level exposure to chlorhexidine may cause a cross-resistance to antibiotics [19]. Hence, the need for developing new antimicrobial agents as alternative therapies to fight oral infections is urgent.

Currently, antimicrobial peptides have captured attention. Researchers have adopted them as a novel and promising antimicrobial approach [20]. Abundant antimicrobial peptides are derived from multicellular organisms and are considered natural antibiotics [13]. Antimicrobial peptides have also been established as the first line of defence against various pathogens, including Gram-positive or -negative microbes, fungi, parasites, and viruses [20]. The mechanism of antimicrobial peptides against pathogens is that net positive-charge peptides can bind directly to the outer bacteria membrane of negatively charged headgroups [21]. Owing to the nonspecific mechanism, antimicrobial peptides have shown great promise, with little to no resistance [22]. Meanwhile, antimicrobial peptides can be effective for microbes that are resistant to conventional antibiotics, and they have low toxicity because their degradation products are natural amino acids [23]. Moreover, antimicrobial peptides can be functionally modified easily with chemical synthesis methods to obtain more small-molecule derivatives [24]. All these advantages make antimicrobial peptides excellent candidates for developing novel anti-infective agents [25], as well as serving as innovative products for immunomodulation and the promotion of wound healing [26]. Consequently, when considering that antimicrobial peptides have great prospects in terms of treating infections, it is relevant to also apply them for oral disease treatment [20].

Naturally, antimicrobial peptides can be found in various organisms, ranging from animals to bacteria, fungi, and plants [27]. Cathelicidin families, one of the most common antimicrobial peptides, are mainly found in mammals [28]. LL-37 is the only cathelicidin in human beings; it is active against various oral Gram-positive and -negative microorganisms due to its amphipathic structure [29]. In addition to its antimicrobial activity, LL-37 is also crucial in immunomodulatory and inflammatory responses [30]. Antimicrobial peptide LR-10, derived from the *Lactobacillus* species, can inhibit the growth of *S. mutans* by forming pores in bacterial membranes [31]. The fungi-derived antimicrobial peptide alamethicin is bacteriocidal against Gram-positive and -negative bacteria [32]. Fa-AMP1 and Fa-AMP2 are novel antimicrobial peptides that are purified from the seeds of buckwheat, and that have antibacterial and antifungal activity [33].

A bibliometric analysis shows a growing global interest in using antimicrobial peptides as functional biomaterials for caries management [34]. Dental caries management philosophy has shifted to minimally invasive dentistry [35,36]. Thus, different bioactive materials, such as biomimetic hydroxyapatite and peptide-based bioactive materials, are introduced to caries management [37,38]. Peptide-based bioactive materials play an important role in inhibiting biofilm growth and remineralising demineralised teeth [39]. For example, GA-KR12, a novel antimicrobial peptide, effectively inhibits *S. mutans* biofilm growth and promotes the remineralisation of artificial enamel and dentin caries [40,41]. Thus, researchers are interested in developing novel antimicrobial peptides for managing oral diseases [42].

Polyphemusin I (PI) is an antimicrobial peptide derived from horseshoe crabs. It can kill bacteria through binding to and by crossing cell membranes, thus rupturing the bacterial membrane [43]. Gallic acid is abundant in fruits and vegetables, and it can accelerate the regeneration of hydroxyapatites due to its pyrogallol group. In addition, gallic acid shows antimicrobial activities [44]. In our previous study, we synthesised a novel peptide (GAPI) by grafting antimicrobial peptide PI to gallic acid. GAPI peptide could be synthesised using the standard fluorenylmethoxycarbonyl solid-phase synthesis method. A multiple-species biofilm study demonstrated that GAPI impact the growth of cariogenic biofilm formation. However, its antimicrobial properties against other common oral pathogens are still unclear. Therefore, the objective of this study was to investigate the antibacterial effects of GAPI on several common oral pathogens.

## 2. Results

### 2.1. Minimum Inhibitory Concentration (MIC) and Minimum Bactericidal Concentration (MBC)

The MIC and MBC of GAPI against *Streptococcus mutans*, *Streptococcus sobrinus*, *Lactobacillus acidophilus*, *Lactobacillus rhamnosus*, *Actinomyces naeslundii*, *Enterococcus faecalis*, *Porphyromonas gingivalis*, and *Actinobacillus actinomycetemcomitans* are summarised in Table 1. 

The MICs of GAPI against *S. mutans* and *S. sobrinus* were 80 μM, whereas the MBCs for these two bacteria were 160 μM and 320 μM, respectively. For *L. acidophilus* and *L. rhamnosus*, the MICs were 40 μM and 20 μM, and the MBCs were 80 μM and 160 μM, respectively. The MICs and MBCs for *A. naeslundii* and *E. faecalis* were 160 μM and 640 μM, respectively. The MICs for *P. gingivalis* and *A. actinomycetemcomitans* were 320 μM and 160 μM, respectively. The MBCs for *P. gingivalis* and *A. actinomycetemcomitans* were 640 μM and 320 μM, respectively. The results indicated that GAPI showed strong antimicrobial activity against cariogenic bacteria. 

### 2.2. Morphology of the Microorganisms

**Figure 1:**
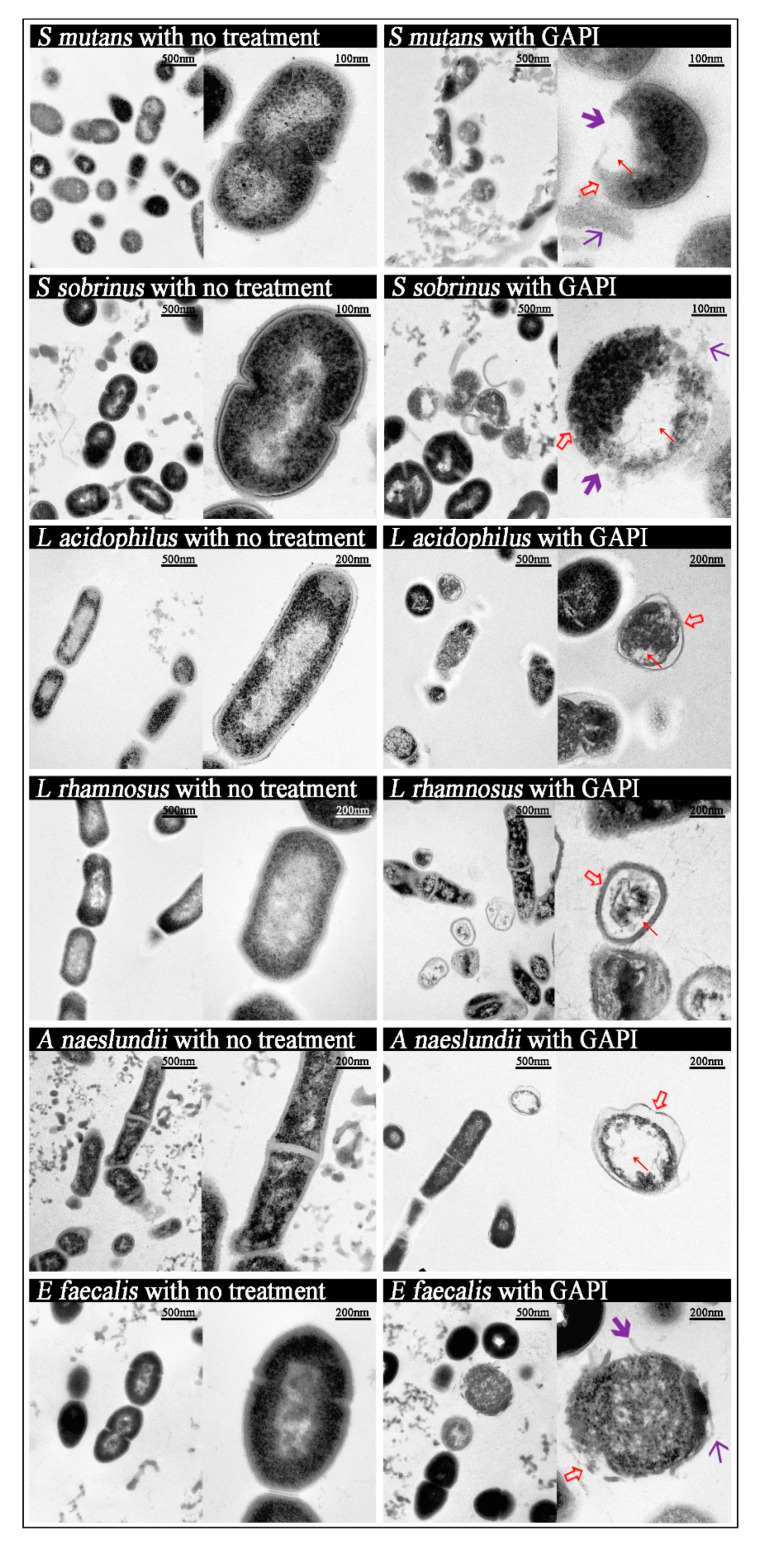
Micrographs of cariogenic pathogens both before and after GAPI treatment. 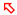 Abnormal cell membrane, 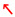 Cytoplasmic clear zone, 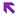 Disrupted cell membrane, 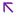 Cytoplasmic content leakage.

Figure 1 represents the morphology of various cariogenic bacteria that were treated with or without GAPI.

*S. mutans* was severely damaged after being treated with GAPI. The *S. mutans* cells lost their normal morphology, with effects including abnormal cell curvatures and irregular cell shapes. The cell wall separated from the cell membrane. In addition, the cells’ cytoplasmic membranes were entirely disrupted, resulting in transparent cytoplasmic zones and the leakage of cytoplasmic contents. 

For GAPI-treated *S. sobrinus*, the morphology changes were similar to GAPI-treated *S. mutans*: the abnormal curvature of cell membranes and irregular cell shapes, clear cytoplasmic zones, the disruption of the cytoplasmic membrane, and the leakage of cytoplasmic contents. 

For *L. acidophilus*, *L. rhamnosus*, and *A. naeslundii,* the typical changes after treatment with GAPI included the abnormal curvature of cell membranes, irregular cell shapes, and cytoplasmic clear zones. 

For *E. faecalis*, compared with untreated bacteria, higher magnification images showed that the bacteria in the GAPI group had abnormal morphological characteristics, including the disruption of the cytoplasmic membrane and the leakage of cytoplasmic contents.

**Figure 2:**
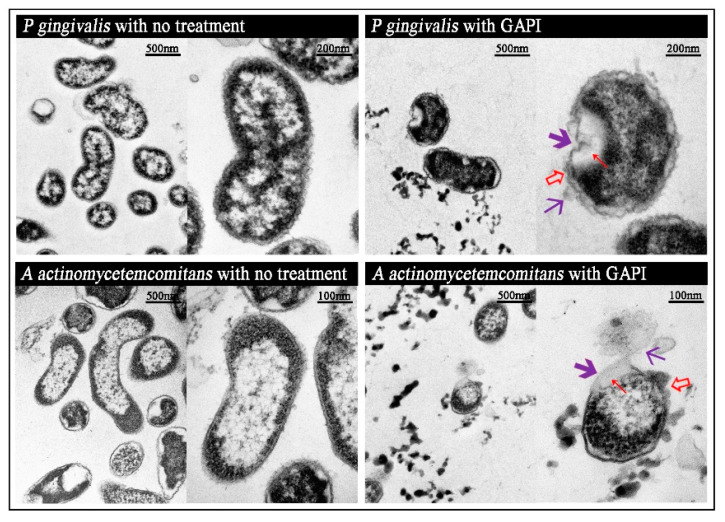
Micrographs of the periodontal pathogens both before and after GAPI treatment. 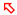 Abnormal cell membrane, 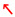 Cytoplasmic clear zone, 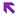 Disrupted cell wall/membrane, 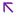 Cytoplasmic content leakage.

Figure 2 represents the morphology of various periodontal-associated bacteria with or without GAPI treatment. For *P. gingivalis* and *A. actinomycetemcomitans*, after being treated with GAPI, the abnormal curvature of cell membranes, irregular cell shapes, and intra-bacterial vacuolisation can be identified. In addition, membrane disruption and the leakage of intracellular components were observed.

## 3. Discussion

Antimicrobial peptides have been studied widely by researchers and are regarded as a new generation of antibiotics due to their broad-spectrum bactericidal activity [45]. In this study, we successfully synthesised GAPI, which consists of the peptide PI as an antimicrobial action domain. Furthermore, gallic acid has been demonstrated to have broad-spectrum antibacterial, antiviral, and antifungal activities [46]. It is a phenolic acid and is easily obtained in large amounts from plants. It has been widely used as an antioxidant additive in food. The addition of gallic acid has potentiated antimicrobials’ effectiveness against various pathogenic bacteria [47]. Accordingly, gallic acid could be applied as a promising compound for new antimicrobial drug development. The antibacterial activity of the synthesised GAPI was investigated against several typical oral pathogenic microorganisms that are frequently found in oral environments.

Cariogenic microbes are essential for caries development. The definition of cariogenic microorganisms includes the following factors: (1) the bacteria have strong bond affinity to the tooth surface; (2) the bacteria can synthesise extracellular and intracellular polysaccharides; (3) the bacteria are acidogenic, transporting and metabolising various carbohydrates; and (4) the bacteria can tolerate acid environments [48]. *Streptococcus*, *Lactobacillus*, and *Actinomyces* species are three common cariogenic microorganisms’ taxa. 

It has been largely accepted that *S. mutans* plays a critical role in biofilm formation, depending on its core attributes [49]. *S. mutans* possesses multiple high-affinity surface adhesins, thereby enabling colonisation even in the absence of sucrose. It can synthesise large quantities of extracellular glucan polymers from sucrose, which is useful in the permanent colonisation of hard surfaces and in forming extracellular polymeric matrices in situ [50]. In addition, *S. mutans* can provide a favourable niche for other bacterial species to colonise in the oral cavity by altering the local environment [51]. Moreover, it has acidogenic characteristics. *S. sobrinus* is another common cariogenic bacterium in the *Streptococcus* taxa. Studies have shown that *S. sobrinus* is more associated with caries’ development progress, especially in early childhood caries [52]. *S. sobrinus* is capable of producing acid and is acid tolerant [53]. Several studies have indicated a significant association between *S. sobrinus* and caries, thereby showing that *S. sobrinus* is more effective in promoting caries than *S. mutans* [54]. Regardless, *S. mutans* and *S. sobrinus* have been implicated as the primary cariogenic microorganisms in biofilm. Therefore, targeting *S. mutans* and *S. sobrinus* growth could be useful in preventing cariogenic biofilm formation.

*Lactobacillus* strains are frequently identified at active carious lesions in adults and children. Among the *Lactobacillus* species found from carious lesions, *L. acidophilus* and *L. rhamnosus* are two dominant microorganisms. *Lactobacillus* species can produce weak acids and tolerate low-pH environments [55]. They are strictly fermentative bacteria and are known for their high capacity for enzyme production. These enzymes enable the *Lactobacillus* species to rapidly break down various carbohydrates into acidic products, at least half of which is lactic acid. In addition, *Lactobacillus* species can grow and remain viable at a lower PH to cope with acid stresses [56]. Unlike *Streptococcus mutans*, which has been well characterised in terms of pathophysiology, the mechanisms of the *Lactobacillus* species still require further investigation.

*A. naeslundii*, a facultative anaerobic Gram-positive bacteria, is related to dental plaque ageing [57]. It can penetrate into dentinal tubules via exposed dentine, thus causing dentin or root caries, and promoting infections of root canal systems [58]. *E. faecalis* is the most commonly isolated bacteria from root canal systems in endodontic infection teeth. It is an anaerobic Gram-positive facultative microorganism that is highly resistant to antimicrobial agents and can survive in very harsh environments, such as low oxygen or poor nutrient supply [7].

*P. gingivalis* and *A. actinomycetemcomitans* are two of the most frequently associated bacteria with periodontitis [59]. *P. gingivalis,* as a keystone pathogen of periodontitis, can produce different kinds of virulence factors, such as lipopolysaccharide, vesicles, gingipains, and fimbriae [60]. These factors destroy not only periodontal tissue directly, but also cause secondary tissue damage by inducing an inflammatory reaction. In addition, *P. gingivalis* forms a dynamic balance and symbiotic relationship with the host, thereby allowing the bacteria to evade the host’s immune reaction. Thus, *P. gingivalis* is regarded as a significant periodontal pathogen that is close to periodontitis’ development, progression, severity, and recurrence [61]. *A. actinomycetemcomitans* is associated with chronic and aggressive periodontitis [62]. It can produce a variety of virulence factors, including endo- and exotoxins. These factors can directly damage host tissues, as well as protect the bacteria from host defences. In addition, *A. actinomycetemcomitans* can impersonate normal epithelial cell functions in order to induce its uptake and to also disseminate into neighbour epithelial cells.

Antimicrobial susceptibility is a key determinant in the process of antimicrobial drug selection, which can be tested via MIC. It is necessary in the application of MIC-guided antimicrobial therapy [63]. According to the results of the present study, GAPI exhibited significant antibacterial efficiency. The MICs and MBCs against eight bacteria were shown to range from 20 to 320 μM and 80 to 640 μM, respectively, which are better than the other peptides from existing studies (MICs and MBCs ranged from 160 to 320 μM and 640 to 1280 μM, respectively) [40].

Furthermore, TEM was used to show bacterial morphology changes after GAPI treatment in order to further understand GAPI’s mechanism. The micrographs revealed that the GAPI disrupted the bacterial membrane, thus causing abnormal membrane curvature, irregular cell shapes, and intra-bacterial vacuolisation, and inducing cytoplasmic components to escape from the microorganism. The mechanism of action begins with GAPI binding to bacteria and then interacting with the cytoplasmic membrane, thereby crossing the cytoplasmic membrane and damaging the membrane integrity. The damage to the integrity of the cell membrane is an important mechanism, by which antibacterial methods deactivate microorganisms. Furthermore, the TEM images indicated that GAPI could damage the bacterial cell structure, causing cytoplasmic content leakage. 

Indeed, this observation is consistent with previous studies showing that positively charged antimicrobial peptides can initially bind to negatively charged phospholipids on the outer leaflet of a bacterial membrane [40]. Most antimicrobial peptides contain hydrophilic and hydrophobic residues at either end. After the initial electrostatic interactions, the antimicrobial peptides accumulate at the surface until reaching a certain concentration. Then, the hydrophobic ends insert into the lipid bilayer, disrupting the bacterial cell membrane and resulting in the leakage of cytoplasmic contents, further resulting in the death of bacteria [21]. Different action models can describe this mechanism, including barrel-stave pore, carpet-like, and toroidal pore models. In addition, antimicrobial peptides can translocate to the inner cytoplasmic leaflet, potentially targeting intracellular components.

In the reaction stage, the specific cationic nature is critical. Studies have shown that there is a correlation between antibacterial activity and charge, as an increasing charge is related to strengthened antibacterial activity. However, too much charge may hinder the antimicrobial activity because the strong interaction of the peptide and lipid head group will inhibit the translocation of antimicrobial peptides into the membrane’s inner leaflet. On the other hand, hydrophobic residues are another feature of antimicrobial peptides. Hydrophobicity determines the degree to which water-soluble antimicrobial peptides can move into the membrane lipid bilayer. Peptides lacking hydrophobic residues typically have poor membrane attachment. However, excessive hydrophobicity can cause cell toxicity and antimicrobial specificity loss [64]. 

It should be noted that negatively charged phospholipids are more commonly found in bacterial cell membranes when compared to neutral mammalian host cell membranes [65,66]. According to the significant difference in their respective bacterial cell envelopes, these bacteria are thus classified as Gram-positive and Gram-negative. Both have similar inner or cytoplasmic membranes. For Gram-negative bacteria, the outer membrane consists of two layers: the inner leaflet of this membrane contains phosphate lipids, while the outer leaflet is composed principally of lipopolysaccharide. Lipopolysaccharide molecules are highly decorated with negatively charged phosphate groups. In comparison, Gram-positive bacteria are surrounded by peptidoglycan layers that are many times thicker than Gram-negative bacteria. Teichoic acids embedded in peptidoglycan are long anionic polymers [67]. Thus, Welling et al. designed an in vivo study to test whether antimicrobial peptides can distinguish microbial cells and host tissues. They indicated that antimicrobial peptides could discriminate between microorganisms and host tissues and also can accumulate at infection sites. Overall, inherent structures or functions of microbial versus host cells contribute to the selective antimicrobial discretion of certain peptides [68]. 

As alternative antibacterial agents, antimicrobial peptides are also known as host defence peptides [69], as they can not only clear the infected bacteria, but also enhance the human immune response. Thus, antimicrobial peptides can selectively kill bacteria without damage to the host cell. In addition, studies have shown that antimicrobial peptides rarely produce microbial resistance because the antimicrobial peptide’s hydrophobic tail can directly enter the bacterial liquid bilayer [66]. The membrane-active mechanism is particularly important when targeting antibiotic-resistant pathogens.

Antimicrobial peptides can be classified into four broad subclasses, including α-helical and β sheets, as well as αβ and non-αβ structures [24]. Moreover, β-hairpin antimicrobial peptides are abundant in animal species and can be isolated in invertebrates and vertebrates. Further, β-hairpin peptides are more active in crossing bacterial cell membranes and accessing intracellular targets [65]. It is noted that small-size β-hairpin antimicrobial peptides have a high resistance to proteolytic degradation [70]. In our study, peptide PI, from the American horseshoe crab *Limulus polyphemus*, is an antimicrobial cell-penetrating peptide with a β-hairpin structure. Additionally, the primary target of β-hairpin antimicrobial peptides is the cellular membrane. Under this premise, the cell-penetrating peptide PI can pass through a cell membrane without interaction with specific receptors. In the present study, the addition of gallic acid did not change the antimicrobial properties of the peptide. Therefore, the new antimicrobial peptide GAPI could be considered a promising alternative antibacterial agent to traditional antibiotics in treating dental diseases.

## 4. Materials and Methods

### 4.1. Peptide Synthesis

GAPI was synthesised using standard fluorenylmethoxycarbonyl synthesis by standard solid-phase peptide synthesis. The GAPI powder was dissolved in sterile deionised water to a specific concentration for study and was stored at −20 °C.

### 4.2. Microorganisms

Eight common oral pathogenic bacterial strains were selected for this study. They are *Streptococcus mutans* ATCC 35668, *Streptococcus sobrinus* ATCC 33478, *Lactobacillus acidophilus* ATCC 9224, *Lactobacillus rhamnosus* ATCC10863, *Actinomyces naeslundii* ATCC 12104, *Enterococcus faecalis* ATCC 29212, *Porphyromonas gingivalis* ATCC 33277, and *Actinobacillus actinomycetemcomitans* ATCC 29523. All the strains were cultured anaerobically.

### 4.3. MIC and MBC

Brain heart infusion (BHI) medium was used for culture of *S. mutans*, *S. sobrinus*, *L. acidophilus*, *L. rhamnosus*, *A. naeslundii*, *E. faecalis*, and *A. actinomycetemcomitans*, whereas p.g. broth was used for culture of *Porphyromonas gingivalis*. The standard dilution method in a 96-well microplate was conducted in order to evaluate the antimicrobial efficacy of GAPI. Each well was filled with 100 μL GAPI dilutions. In addition, serial twofold dilutions in concentrations ranging from 1280 μM to 1.25 μM were prepared. A 10 μL bacterial culture (10^6^ CFU/mL) was added. Chlorhexidine was used as positive control, and medium was used as negative control. The plates were then anaerobically incubated at 37 °C for 24 h. The absorbance was measured at a wavelength of 660 nm in order to analyse the growth of microorganisms. The MIC value was defined as the lowest concentration at which no visible growth was seen in the clear well. After the MIC determination, 10 μL fluid from each well, which showed no visible bacterial growth, was pipetted and seeded on blood agar, which were then put into an anaerobic incubator at 37 °C for 48 h. The MBC endpoint was the lowest concentration at which 99.9% of the bacterial population was killed, which thus means the absence of bacteria.

### 4.4. Morphology of the Microorganisms

Bacteria morphology was observed using a transmission electron microscope (TEM, Philips CM100). GAPI was added to a bacterial culture of 10^8^ CFU/mL, and the bacteria were harvested after incubating at 37 °C for 18 h. The semi-thin sections of cell were contained in grids and examined with the TEM.

## 5. Conclusions

This laboratory study showed that the novel antimicrobial peptide GAPI has promising antibacterial effects against common cariogenic and periodontal pathogens. It can also serve as an alternative to antibiotics in terms of managing dental infection.

## Figures and Tables

**Table 1:** The minimum inhibitory concentration (MIC) and minimum bactericidal concentration (MBC) of GAPI against common American Type Culture Collection (ATCC) oral pathogens.

Bacteria	ATCC	MIC (μM)	MBC (μM)
*Actinobacillus actinomycetemcomitans*	29523	160	320
*Actinomyces naeslundii*	12104	160	640
*Enterococcus faecalis*	29212	160	640
*Lactobacillus acidophilus*	9224	40	80
*Lactobacillus rhamnosus*	10863	20	160
*Porphyromonas gingivalis*	33277	320	640
*Streptococcus mutans*	35668	80	160
*Streptococcus sobrinus*	33478	80	320

## Data Availability

The data are contained within the article.
